# Disease-Causing *TIMP3* Variants and Deep Phenotyping of Two Czech Families with Sorsby Fundus Dystrophy Associated with Novel p.(Tyr152Cys) Mutation

**DOI:** 10.3390/ijms25073744

**Published:** 2024-03-27

**Authors:** Andrea Vergaro, Monika Pankievic, Jana Jedlickova, Lubica Dudakova, Marie Vajter, Michel Michaelides, Martin Meliska, Pavel Nemec, Daniela Babincova, Bohdan Kousal, Petra Liskova

**Affiliations:** 1 Department of Paediatrics and Inherited Metabolic Disorders, First Faculty of Medicine, Charles University and General University Hospital in Prague, 121 08 Prague, Czech Republic; andrea.vergaro@vfn.cz (A.V.); jana.moravikova@seznam.cz (J.J.); lubica.dudakova@lf1.cuni.cz (L.D.); marie.vajter@vfn.cz (M.V.); 2 Department of Ophthalmology, First Faculty of Medicine, Charles University and General University Hospital in Prague, 128 08 Prague, Czech Republic; martin.meliska@vfn.cz (M.M.); bohdan.kousal@vfn.cz (B.K.); 3 UCL Institute of Ophthalmology, University College London and Moorfields Eye Hospital, London EC1V 9EL, UK; michel.michaelides@ucl.ac.uk; 4 Department of Ophthalmology, First Faculty of Medicine and Military University Hospital Prague, 162 00 Prague, Czech Republic; pavel.nemec@uvn.cz; 5 Laboratory of Molecular Biology, AGEL, 741 01 Nový Jíčín, Czech Republic; daniela.babincova@lab.agel.cz

**Keywords:** Sorsby fundus dystrophy, choroidal neovascular membrane, TIMP3, pathogenic variants, optical coherence tomography angiography

## Abstract

We aim to report the ocular phenotype and molecular genetic findings in two Czech families with Sorsby fundus dystrophy and to review all the reported *TIMP3* pathogenic variants. Two probands with Sorsby fundus dystrophy and three first-degree relatives underwent ocular examination and retinal imaging, including optical coherence tomography angiography. The DNA of the first proband was screened using a targeted ocular gene panel, while, in the second proband, direct sequencing of the *TIMP3* coding region was performed. Sanger sequencing was also used for segregation analysis within the families. All the previously reported *TIMP3* variants were reviewed using the American College of Medical Genetics and the Association for Molecular Pathology interpretation framework. A novel heterozygous variant, c.455A>G p.(Tyr152Cys), in *TIMP3* was identified in both families and potentially de novo in one. Optical coherence tomography angiography documented in one patient the development of a choroidal neovascular membrane at 54 years. Including this study, 23 heterozygous variants in *TIMP3* have been reported as disease-causing. Application of gene-specific criteria denoted eleven variants as pathogenic, eleven as likely pathogenic, and one as a variant of unknown significance. Our study expands the spectrum of *TIMP3* pathogenic variants and highlights the importance of optical coherence tomography angiography for early detection of choroidal neovascular membranes.

## 1. Introduction

Sorsby fundus dystrophy (SFD, MIM #136900) is an ultra-rare autosomal-dominant disorder with complete penetrance and an estimated prevalence of 1/220,000 [1]. SFD is characterized by the accumulation of protein/lipid deposits under the retinal pigment epithelium (RPE), referred to as drusen [1,2]. Histopathological characterization has been quite limited in SFD because of the disease rarity. According to the available studies, thickening of Bruch’s membrane is an invariable feature. The histological hallmark of SFD appears to be confluent amorphous deposits located between the inner collagenous layer of Bruch’s membrane and the basement membrane of the RPE [2]. 

The disease is caused by pathogenic variants in the tissue inhibitor of metalloproteinase 3 (*TIMP3*) gene. The encoded TIMP3 protein inhibits not only a broad array of matrix metalloproteinases but also several members of the disintegrin and metalloproteinase domain family and thus contributes to controlling diverse processes mediated by proteolysis [3]. In the outer retina, TIMP3 protein is thought to have an important role in regulating the thickness of Bruch’s membrane by inhibiting its degradation from local metalloproteinases and in the inhibition of angiogenesis via blockage of vascular endothelial growth factor (VEGF) signaling [1,4,5]. The mechanism responsible for disease onset in the presence of pathogenic *TIMP3* gene variants is still poorly understood. It has been hypothesized that SFD could be caused by the abnormal accumulation of TIMP3 protein in Bruch’s membrane and/or indirectly by dysregulation of the extracellular matrix [1].

The disease usually manifests with dark adaptation problems or a sudden loss of visual acuity due to subfoveal choroidal neovascularization (CNV), typically in the third or fourth decade of life [1]. In the initial stages, drusen-like deposits throughout the fundus are found. As the disease progresses, macular edema, exudates, retinal hemorrhages, or RPE detachment may develop together with CNV. Progressive bilateral outer retinal atrophy is another hallmark of the disease. Advanced SFD is characterized by macular scarring and/or severe atrophy, often associated with previous recurrent CNV [1,2]. Currently, there is no effective treatment for SFD; however, CNV as a secondary complication can be managed with prompt intravitreal anti-VEGF therapy [2,6,7]. 

Optical coherence tomography angiography (OCTA) is a quick non-invasive imaging technique constructing a map of blood flow in different retinal layers [8]. Presumably, due to the rarity of the disease, only a few reports describing OCTA findings in SFD have been published to date [9,10,11,12,13]. 

One study suggested that SFD may involve not only the retina but also extraocular tissues. Late-onset pulmonary disease manifesting as severe emphysema, despite no history of smoking or asthma, was observed in four affected members from one family with SFD. Another affected individual with SFD from an unrelated family had mild to marked bronchial wall thickening with mild cylindrical bronchiectasis [14].

In this study, we report a novel disease-causing variant in *TIMP3*. In addition, deep phenotyping allowed us to document an early CNV using OCTA. We also review *TIMP3* variants reported as disease-causing by applying the classification of the American College of Medical Genetics and Genomics (ACMG) and the Association for Molecular Pathology (AMP). 

## 2. Results

**Figure 1:**
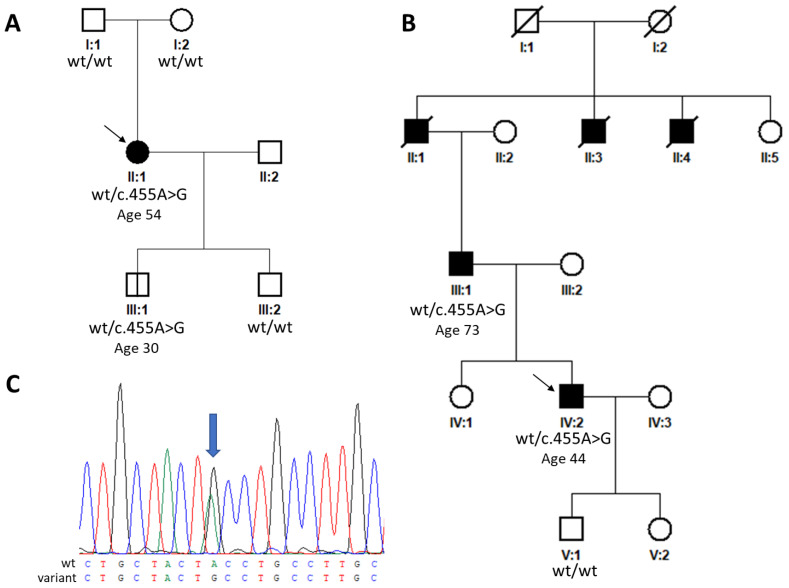
Pedigrees and sequence chromatogram. (**A**) Pedigrees of family 1, (**B**) family 2, and (**C**) sequence chromatogram of the detected heterozygous *TIMP3* variant (blue arrow) altering a tyrosine to cysteine NM_000362.5:c.455A>G p.(Tyr152Cys). Black squares and circles indicate clinically affected individuals, white symbols represent clinically unaffected individuals. Arrows indicate probands. Abbreviation: wt, wild type.

**Figure 2:**
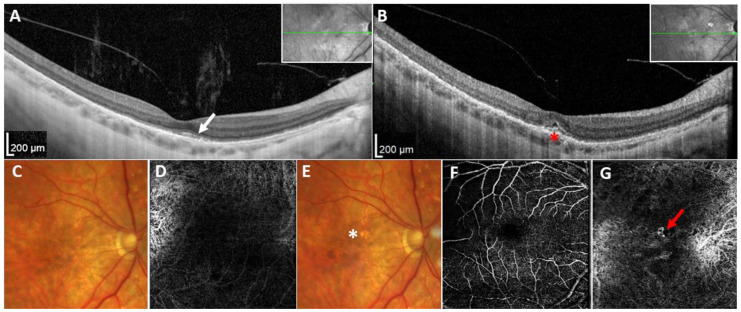
Retinal imaging in the proband from family 1 documenting development of CNV in the right eye. (**A**) Baseline SD-OCT horizontal scans through the macula (cross-sectional plane is provided in the insert) performed at the age of 54 years, showing small subfoveolar drusen, irregularities within the photoreceptors layer (white arrow), and a partial vitreous detachment. (**B**) SD-OCT scan performed 6 months later; note a small area of neuroretinal detachment in the central foveolar region (red asterisk). (**C**) Baseline fundus photography documenting irregular pigment distribution and drusen in the macular and paramacular area, and (**D**) OCTA image within avascular complex with no signs of neovascularization. (**E**) Fundus photography taken after CNV development; only subtle pigmentary changes in the macular can be recognized (white asterisk), and (**F**) OCTA scan of the deep vascular and (**G**) avascular complex demonstrating the presence of a subfoveolar CNV (red arrow). Abbreviations: CNV, choroidal neovascularization; SD-OCT, spectral domain optical coherence tomography; OCTA, optical coherence tomography angiography.

The proband from family 1 (individual II:1, Figure 1A) had a personal history of nyctalopia from the age of 40. She was referred to the Department of Ophthalmology, First Faculty of Medicine, Charles University and General University Hospital in Prague for examination when aged 54 years after diagnostic next-generation sequencing (NGS) screening, which revealed a heterozygous variant, c.455A>G p.(Tyr152Cys) (Figure 1C), in *TIMP3* considered at that time to be a VUS (variant of uncertain significance). Her best corrected visual acuity (BCVA) was 0.8 bilaterally, and fundus examination detected the presence of drusen-like deposits and diffuse chorioretinal thinning in both eyes (Figure 2C). Spectral domain optical coherence tomography (SD-OCT) examination of the macula revealed focal disruption of the RPE and the photoreceptor layer, while fundus autofluorescence (FAF) demonstrated hyperautofluorescent and hypoautofluorescent spots at the macula (Figure 2A).

Six months later, the proband experienced deterioration of vision in the right eye over a 3-week period, and her BCVA decreased to 0.1, with a positive Amsler grid test for metamorphopsia. BCVA in the left eye remained stable. Biomicroscopic examination of the fundus showed the presence of a pigment deposition in the macular area (Figure 2E), while SD-OCT imaging suggested a type 2 CNV (i.e., passing through the RPE and located above the RPE in the subretinal space) [15] with serous detachment of the neuroretina (Figure 2B). OCTA localized the CNV within the avascular complex layer. The widest measured subfoveolar extension of the CNV covered an area of about 0.16 mm^2^ and was localized in the inferonasal section of the fovea (Figure 2G). After CNV detection, the patient was treated elsewhere with anti-VEGF intravitreal injections.

Her older son (individual III:1, Figure 1A), examined when 30 years old, was completely asymptomatic, with a BCVA of 1.0 in both eyes. Biomicroscopic examination of the fundus and SD-OCT, including OCTA of the macular zone, revealed no pathology (Supplementary Figure S1). Ophthalmic examination with detailed investigation for changes within the macular region using OCT and OCTA has been performed yearly. No pathological findings or changes in measurable retinal parameters (e.g., foveal avascular zone area, central retinal thickness, and choroidal thickness) have been identified up to the date of the manuscript submission when aged 33 years.

The general health of both the proband and her son was otherwise unremarkable, with the only condition present in the medical records being an oligosymptomatic form of osteoarticular Bechterew disease in the mother. Personal history was negative for bronchopulmonary disease in both subjects. The second son of the proband was not available for clinical examination. He reported to be free of any vision problems. The parents could not be examined because of their advanced age and limited mobility. Both reported no major visual problems. Consistently targeted Sanger sequencing for the presence of c.455A>G was negative in both individuals, as shown in Figure 1A.

**Figure 3:**
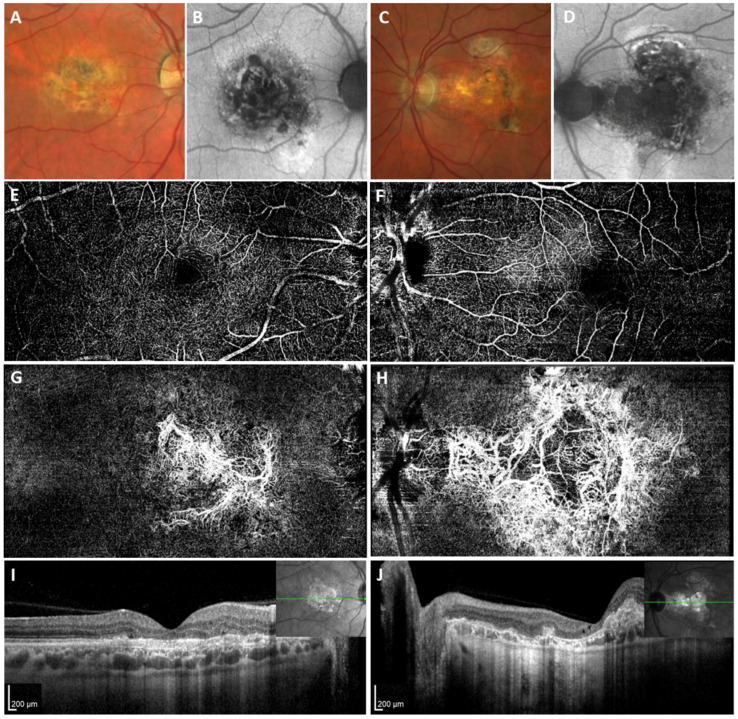
Retinal imaging in the proband from family 2. (**A**) Fundus photography of the right eye and (**C**) left eye showing severe atrophy of the whole macular region with patchy pigmentation. (**B**,**D**) Central macular hypoautofluorescence dominates in FAF images. (**E**,**F**) OCTA scans demonstrate no lesion within the deep vascular complex, while an extensive presumably long-lasting CNV is identified bilaterally in the avascular complex layer (**G**,**H**), corresponding to complete loss of the external retinal layers as demonstrated by transversal SD-OCT scans passing through the fovea (**I**) in the right and (**J**) left eye. Abbreviations: CNV, choroidal neovascularization; FAF, fundus autofluorescence; SD-OCT, spectral domain optical coherence tomography; OCTA, optical coherence tomography angiography.

The proband from family 2 (individual IV:2, Figure 1B) experienced metamorphopsia and deterioration of BCVA due to CNV in the right eye at the age of 37 and in the left eye when aged 39 years. The CNV in the right eye was managed elsewhere with photodynamic therapy following five applications of bevacizumab over one year and thirteen applications of ranibizumab over 7 years. The left eye had a history of five bevacizumab intravitreal injections. When examined by the authors, aged 44, the BCVA was 0.2 in the right eye and 0.08 in the left eye. Biomicroscopic evaluation of the fundus, SD-OCT, and OCTA examination documented the presence of fibrovascular macular scarring in both eyes (Figure 3). 

The family history revealed that the father of the proband experienced severe metamorphopsia at the age of 45 years. When examined at the age of 73 years, his BCVA was 0.1 bilaterally. There was dense fibrovascular scarring in both macular regions, with complete vascularization of the avascular layer bilaterally and total loss of the physiological architecture of the retinal layers on SD-OCT and OCTA (Supplementary Figure S2). The family history indicated that the deceased grandfather of the proband as well as two granduncles were also likely afflicted with SFD. The proband’s only sister reported to be free from any symptoms related to possible macular disease and declined the possibility of preventive screening of the detected pathogenic variant, while mutation analysis in the proband’s 13-year-old daughter was recommended only after counselling with a clinical geneticist and has not been performed yet. Sanger sequencing confirmed the absence of the pathogenic variant in the proband’s 22-year-old son, who accordingly reported no kind of vision impairment. 

The general health of the proband from family 2 was unremarkable, while the 73-year-old father had a history of type 2 diabetes mellitus and arterial hypertension with chronic ischemic heart disease.

A novel *TIMP3* variant, c.455A>G p.(Tyr152Cys), was identified in both families. In family 1, the disease-causing variant occurred most likely de novo as neither of the proband’s parents carried the change, which was supported by paternity and maternity testing (Figure 1A). In addition, the variant was also observed in a 33-year-old asymptomatic son. Segregation analysis in family 2 showed that both the proband and his affected father harbored the sequence variant (Figure 1B), while one asymptomatic son tested negative. 

Based on the available evidence, the c.455A>G p.(Tyr152Cys) variant was classified as pathogenic, meeting the following ACMG/AMP criteria: PM2_Moderate (no frequency in population databases), PS4_Supporting (prevalence in affected individuals is increased), PP3_Supporting (computational prediction tools support a deleterious effect on the gene), PP4_Strong (patient’s phenotype or family history is highly specific for a disease with a single genetic etiology), PM1_Moderate (located in a mutational hot spot and/or functional domain), and PS2 (de novo; paternity and maternity confirmed).

Including this study, 23 heterozygous variants in *TIMP3* have been reported in patients presenting with retinal disease. Application of ACMG/AMP criteria adjusted for *TIMP3* denoted eleven as pathogenic, eleven as likely pathogenic, and one as a VUS (Table 1; Supplementary Table S1).

## 3. Discussion

Herein, we report two Czech families with SFD carrying a novel pathogenic variant, c.455A>G p.(Tyr152Cys), in *TIMP3*. In addition, using OCTA, we have visualized early-stage CNV in one eye, highlighting the utility of this modality. 

Only six cases with SFD have been investigated using OCTA previously [9,10,11,12,13,35]. The development of CNV in SFD is a typical feature of the disease, requiring careful monitoring after 30 years of age. Although fluorescein angiography is considered by some to be the gold standard for confirmation and analysis of active CNV, it is an invasive procedure associated with several albeit uncommon possible complications, which make its use unsuitable for repeated application in cases with no symptoms [36]. As OCTA is non-invasive, it can be extremely helpful in high-risk subjects such as carriers of *TIMP3* pathogenic mutations as a screening tool to identify early-stage CNV, which is important for timing of anti-VEGF treatment and improved outcomes [7,37]. In our experience, fundus examination can be uninformative during this stage. 

One individual aged 33 years at the time of the last examination did not show any signs of SFD despite being a heterozygous carrier of the pathogenic variant. This is, however, not surprising given that onset of the disease occurred in all three affected individuals reported in this study in their fourth or fifth decade of life. 

SFD may pose a diagnostic challenge due to phenotypic variability and late manifestation. During the evaluation process, we initially considered the c.455A>G observed in family 1 as a VUS. The development of CNV, as well as identification of the variant in a second family with SFD, provided additional support to classify the change as pathogenic. When fully manifested, SFD clinical signs are highly specific, allowing for direct screening of *TIMP3* as the only known disease-causing gene. The proband from family 1 had an earlier stage of SFD upon initial clinical examination, not enabling specific genotype correlation; in addition, there was no family history of disease, suggesting a likely recessive inheritance pattern. For these reasons, her DNA sample was analyzed using a broad gene panel, contrary to the proband from family 2, who only underwent direct sequencing of *TIMP3*.

Pathogenic variants in *TIMP3* have also been reported to possibly be associated with chronic obstructive pulmonary disease [14]; in this study, however, all the mutation carriers denied having any bronchopulmonary problems.

As several *TIMP3* reference sequence identifiers have been used in the literature, we have reviewed all the reported variants in the context of canonical transcript NM_000362.5. We have also suggested gene-specific criteria to apply the ACMG/AMP guidelines and used them for *TIMP3* variant pathogenicity assessment. The limitation of the current study is that consensus was only reached among the authors of this manuscript and the suggested gene-specific criteria may be altered later by a larger group of experts.

Out of the 23 *TIMP3* variants previously reported to be retinal disease-causing, c.499G>A p.(Asp167Asn) did not meet the ACMG/AMP criteria for pathogenic/likely pathogenic. As for pathogenic/likely pathogenic variants, most are located in exon 5, and 16 involve cysteine residue (introduction or loss of a cysteine). The novel *TIMP3* mutation described herein also results in changing an amino acid to cysteine, further supporting the hypothesis that mutant *TIMP3* proteins with unpaired cysteines form abnormal disulfide-bonded dimers and aggregate, leading to disturbed extracellular matrix remodeling of Bruch’s membrane [23,24,28,38,39,40,41,42].

## 4. Materials and Methods

### 4.1. Participants and Clinical Examination

Detailed ocular examination was performed in the Department of Ophthalmology, First Faculty of Medicine, Charles University and General University Hospital in Prague. BCVA using Snellen charts (shown in decimal values) and intraocular pressure were measured. Macular architecture was assessed with SD-OCT (Spectralis, Heidelberg Engineering GmbH, Heidelberg, Germany). Fundus photography and FAF imaging were acquired using an ultra-widefield camera (Clarus 700, Carl Zeiss Meditec AG, Jena, Germany).

OCTA was performed using the Spectralis OCT2 Module. Angiography images with a field of view of 20° × 20° (~5.8 mm × 5.8 mm) or 30° × 15° (~8.8 mm × 4.4 mm) centered on the fovea showing the superficial vascular complex (SVC), the deep vascular complex (DVC), and the avascular complex were collected for analysis. The images were generated by automated layer segmentation using the software of the OCT instrument (version 7.0.1). SVC was defined as the area between the internal limiting membrane and inner plexiform layer (IPL), DVC as the area between the IPL and the outer plexiform layer (OPL), while the avascular complex was defined as the area spanning from OPL to basal membrane [43].

### 4.2. Molecular Genetic Analysis

Genomic DNA was isolated from peripheral blood using Gentra Puregene™ Blood Kit (Qiagen, Hilden, Germany) or saliva samples using Oragene Saliva Collection and DNA extraction kit (Genotek, Ottawa, ON, Canada) according to the manufacturer’s instructions. 

In the proband from family 1, NGS using a panel comprising 548 genes implicated in ocular disorders was conducted (Supplementary Materials). Library preparation was performed with Kapa Hyper Prep Kit (Roche, Basel, Switzerland) and the KAPA UDI Primer Mixes (Roche) for indexing. Enrichment was conducted with designed target enrichment probes (Roche). Sequencing was performed on the MiniSeq platform (Illumina, San Diego, CA, USA). Paternity and maternity testing was carried out with a set of 16 forensic markers [44].

In proband 2, the entire coding region of *TIMP3* and flanking introns were prioritized based on specific phenotype and bidirectionally analyzed by conventional Sanger sequencing using previously described primers [45]. Direct sequencing was also used to verify the presence of presumed causal variant detected by NGS and for targeted screening in first-degree relatives. NCBI Reference Sequence NM_000362.5 was used. 

### 4.3. Review of TIMP3 Variants 

Variants in *TIMP3* previously reported to be associated with SFD or a retinal dystrophy phenotype were reviewed and aligned to NM_000362.5. If the original work used a different reference sequence, variant description was extrapolated. If no reference sequence was provided but sequence chromatogram with the variant was shown, we plotted the change in the context of NM_000362.5. If no reference sequence or sequence chromatogram were provided in the original publication, we extrapolated the change at DNA level based on literature data published at the time. If this was the case, we checked other transcripts used in reports on *TIMP3* variants for consistency with the nucleotide and protein change.

The ACMG along with the AMP published their guidelines for sequence variant interpretation in 2015. Since then, several Variant Curation Expert Panels were established to define application of the ACMG/AMP guidelines in specific genes or diseases. As none of them deals with *TIMP3,* we have created ACMG/AMP-specific criteria following published recommendations on selected genes causing rare autosomal-dominant disorders with high degree of penetrance (Table 2) [46,47]. PP5 criterion was not used as recently recommended [48]. The final variant scoring to one of the five categories (pathogenic, likely pathogenic, variant of uncertain significance (VUS), likely benign, and benign) was performed using the Franklin platform (https://franklin.genoox.com (accessed on 10 February 2024)).

## 5. Conclusions

In summary, this is the first report regarding SFD in the Czech population, which highlights the usefulness of molecular genetic testing and the necessity of deep genotype–phenotype correlations. The novel pathogenic variant and clinical findings described in this study continue to broaden the molecular genetic knowledge on SFD and reemphasize the importance of considering SFD in differential diagnosis regarding patients with unspecified macular dystrophy.

Individuals with pathogenic/likely pathogenic *TIMP3* variants should be instructed to regularly self-examine with an Amsler grid and if possible regularly followed by using OCTA in order to detect CNV early, which can then be managed with prompt anti-VEGF treatment. In addition to CNV identification, serial OCT and OCTA examination in asymptomatic *TIMP3* pathogenic variant carriers could be an invaluable tool to discover early disease markers and to shed light on the pathophysiological mechanisms involved in disease progression. Unfortunately, we did not identify any presymptomatic changes using these methods. In this context, we must take into account the short duration of the total follow-up period and the relatively young age of the examined subjects. 

## Figures and Tables

**Table 1:** Summary and classification of *TIMP3* variants reported as disease-causing in the heterozygous state.

Description		ACMG/AMP Classification	Reported Phenotype	References
DNA	Protein			
c.29T>A	p.(Leu10His)	Likely pathogenic	Early-onset maculopathy without CNV	[16]
c.34G>C	p.(Gly12Arg)	Likely pathogenic	Early-onset maculopathy without CNV	[16]
c.70T>C	p.(Cys24Arg)	Likely pathogenic	SFD (no clinical description)	[17]
c.113C>G	p.(Ser38Cys)	Pathogenic	SFD	[17,18,19,20]
c.410A>G	p.(Tyr137Cys)	Likely Pathogenic	Retinitis pigmentosa-like, drusen	[21]
c.439-2dup	p.?	Likely Pathogenic	SFD	[22]
c.452A>G	p.(Tyr151Cys)	Likely Pathogenic	SFD (no clinical description)	[17]
c.455A>G	p.(Tyr152Cys)	Pathogenic	SFD	Current study
c.484G>A	p.(Glu162Lys)	Likely pathogenic	SFD	[23]
c.484G>T	p.(Glu162*)	Likely pathogenic	SFD	[24,25]
c.499G>A	p.(Asp167Asn)	VUS	MD vs. SFD (no clinical description)	[26]
c.521A>G	p.(Tyr174Cys)	Pathogenic	SFD	[6]
c.530A>G	p.(Tyr177Cys)	Pathogenic	SFD	[6]
c.536C>G	p.(Ser179Cys)	Pathogenic	SFD	[24,27]
c.542A>G	p.(His181Arg)	Pathogenic	SFD	[28]
c.545A>G	p.(Tyr182Cys)	Pathogenic	SFD	[6,29]
c.565G>T	p.(Gly189Cys)	Pathogenic	SFD	[24,30]
c.568G>T	p.(Gly190Cys)	Pathogenic	SFD	[31]
c.572A>G	p.(Tyr191Cys)	Likely pathogenic	SFD	[32]
c.577A>T	p.(Ser193Cys)	Likely pathogenic	Retinitis pigmentosa-like, drusen	[33]
c.584A>G	p.(Tyr195Cys)	Pathogenic	SFD	[31]
c.594G>T	p.(Trp198Cys)	Likely pathogenic	SFD (no clinical description)	[17]
c.610A>T	p.(Ser204Cys)	Pathogenic	SFD	[32,34]

NM_000362.5 was used as the reference sequence. Abbreviations: VUS, variant of uncertain significance; CNV, choroidal neovascular membrane; SFD, Sorsby fundus dystrophy; MD, macular dystrophy.

**Table 2:** ACMG/AMP criteria specific to *TIMP3*, which were used to score variants.

Pathogenic Criteria		ACMG/AMP Criteria	Strong	Moderate	Supporting
Population data	PM2	Absent in population databases		gnomAD non-founder subpopulations frequency 0.0%	gnomAD non-founder subpopulations frequency <0.0001%
	PS4	The prevalence of the variant in affected individuals is significantly increased compared with the prevalence in controls	≥5 observations	3–4 observations	2nd independent occurrence
Computational and predictive data	PP3	Multiple lines of computational evidence support a deleterious effect on the gene or gene product	REVEL score > 0.932	REVEL score (0.773–0.932)	REVEL score (0.644–0.772) or SpliceAI score > 0.5 for splicing variants
Phenotype	PP4	Patient’s phenotype or family history is highly specific for a disease with a single genetic aetiology	Clinical diagnosis of SFD with consistent phenotype description	Clinical diagnosis of SFD without phenotype description	Clinical diagnosis of macular dystrophy or retinitis pigmentosa without CNV and/or drusen with phenotype description
Functional data	PS3	Well-established in vitro or in vivo functional studies supportive of a damaging effect on the gene or gene product			Studies support functional impact
	PM1	Located in a mutational hot spot and/or functional domain without benign variation		Variant leads to the introduction or loss of a cysteine	
Segregation data	PP1	Co-segregation with disease in multiple affected family members in a gene definitively known to cause the disease	Co-segregation with disease ≥7 meiosis	Co-segregation with the disease in 5–6 meiosis	Co-segregation with the disease in 3–4 meiosis
De novo data	PS2	De novo in a patient with disease and no family history	De novo in a patient with phenotype consistency; no family history and both maternity and paternity are confirmed		
	PM6			De novo in a patient with phenotype consistency; no family history and both maternity and paternity are assumed	

If PM1 was met with a Moderate strength, and PP3 was met with a Strong strength (PP3_Strong), the total evidence used was only PP3_Strong. PM1_Moderate was applied as cysteines are important for TIMP3 function [49,50]. PM2_Supporting was applied as individuals within gnomAD are generally free from severe Mendelian childhood-onset disease, whereas SFD is typically late-onset.

## Data Availability

Data are available from the corresponding author upon reasonable request.

## Supplementary Materials

The supporting information can be downloaded at https://www.mdpi.com/article/10.3390/ijms25073744/s1.

## References

[1] Christensen D.R.G., Brown F.E., Cree A.J., Ratnayaka J.A., Lotery A.J. (2017). Sorsby fundus dystrophy—A review of pathology and disease mechanisms. Exp. Eye Res..

[2] Anand-Apte B., Chao J.R., Singh R., Stohr H. (2019). Sorsby fundus dystrophy: Insights from the past and looking to the future. J. Neurosci. Res..

[3] Arpino V., Brock M., Gill S.E. (2015). The role of TIMPs in regulation of extracellular matrix proteolysis. Matrix Biol..

[4] Qi J.H., Ebrahem Q., Moore N., Murphy G., Claesson-Welsh L., Bond M., Baker A., Anand-Apte B. (2003). A novel function for tissue inhibitor of metalloproteinases-3 (TIMP3): Inhibition of angiogenesis by blockage of VEGF binding to VEGF receptor-2. Nat. Med..

[5] Kamei M., Hollyfield J.G. (1999). TIMP-3 in Bruch’s membrane: Changes during aging and in age-related macular degeneration. Investig. Ophthalmol. Vis. Sci..

[6] Gliem M., Muller P.L., Mangold E., Holz F.G., Bolz H.J., Stohr H., Weber B.H., Charbel Issa P. (2015). Sorsby Fundus Dystrophy: Novel Mutations, Novel Phenotypic Characteristics, and Treatment Outcomes. Investig. Ophthalmol. Vis. Sci..

[7] Tsokolas G. (2022). Sorsby fundus dystrophy (SFD): A narrative review. Medicine.

[8] de Carlo T.E., Romano A., Waheed N.K., Duker J.S. (2015). A review of optical coherence tomography angiography (OCTA). Int. J. Retin. Vitr..

[9] Mohla A., Khan K., Kasilian M., Michaelides M. (2016). OCT angiography in the management of choroidal neovascular membrane secondary to Sorsby fundus dystrophy. BMJ Case Rep..

[10] Spaide R.F. (2022). Treatment of Sorsby fundus dystrophy with anti-tumor necrosis factor-alpha medication. Eye.

[11] Tsokolas G., Almuhtaseb H., Lotery A. (2020). Evaluation of Pro-re-Nata (PRN) and Treat and Extend Bevacizumab treatment protocols in Sorsby Fundus Dystrophy. Eur. J. Ophthalmol..

[12] Spaide R.F. (2022). Long-Term Visual Acuity Preservation in Sorsby Fundus Dystrophy with Corticosteroid Treatment. Retin. Cases Brief Rep..

[13] Hess K., Raming K., Gliem M., Charbel Issa P., Herrmann P., Holz F.G., Pfau M. (2022). Choriocapillaris Flow Signal Impairment in Sorsby Fundus Dystrophy. Ophthalmologica.

[14] Meunier I., Bocquet B., Labesse G., Zeitz C., Defoort-Dhellemmes S., Lacroux A., Mauget-Faysse M., Drumare I., Gamez A.S., Mathieu C. (2016). A new autosomal dominant eye and lung syndrome linked to mutations in TIMP3 gene. Sci. Rep..

[15] Bloch S.B. (2013). Implementation studies of ranibizumab for neovascular age-related macular degeneration. Acta Ophthalmol..

[16] Guan B., Huryn L.A., Hughes A.B., Li Z., Bender C., Blain D., Turriff A., Cukras C.A., Hufnagel R.B. (2022). Early-Onset TIMP3-Related Retinopathy Associated with Impaired Signal Peptide. JAMA Ophthalmol..

[17] Bakall B., Sohn E.H., Riley J., Brack D., Stone E.M. (2014). Novel mutations and change of nomenclature for pathogenic variants in the TIMP3 gene causing Sorsby fundus dystrophy. Investig. Ophthalmol. Vis. Sci..

[18] Naessens S., De Zaeytijd J., Syx D., Vandenbroucke R.E., Smeets F., Van Cauwenbergh C., Leroy B.P., Peelman F., Coppieters F. (2019). The N-terminal p.(Ser38Cys) TIMP3 mutation underlying Sorsby fundus dystrophy is a founder mutation disrupting an intramolecular disulfide bond. Hum. Mutat..

[19] Schoenberger S.D., Agarwal A. (2013). A novel mutation at the N-terminal domain of the *TIMP3* gene in Sorsby fundus dystrophy. Retina.

[20] Warwick A., Gibson J., Sood R., Lotery A. (2016). A rare penetrant TIMP3 mutation confers relatively late onset choroidal neovascularisation which can mimic age-related macular degeneration. Eye.

[21] DeBenedictis M.J., Gindzin Y., Glaab E., Anand-Apte B. (2020). A novel *TIMP3* mutation associated with a retinitis pigmentosa-like phenotype. Ophthalmic Genet..

[22] Tabata Y., Isashiki Y., Kamimura K., Nakao K., Ohba N. (1998). A novel splice site mutation in the tissue inhibitor of the metalloproteinases-3 gene in Sorsby’s fundus dystrophy with unusual clinical features. Hum. Genet..

[23] Saihan Z., Li Z., Rice J., Rana N.A., Ramsden S., Schlottmann P.G., Jenkins S.A., Blyth C., Black G.C., McKie N. (2009). Clinical and biochemical effects of the E139K missense mutation in the *TIMP3* gene, associated with Sorsby fundus dystrophy. Mol. Vis..

[24] Langton K.P., McKie N., Curtis A., Goodship J.A., Bond P.M., Barker M.D., Clarke M. (2000). A novel tissue inhibitor of metalloproteinases-3 mutation reveals a common molecular phenotype in Sorsby’s fundus dystrophy. J. Biol. Chem..

[25] Clarke M., Mitchell K.W., Goodship J., McDonnell S., Barker M.D., Griffiths I.D., McKie N. (2001). Clinical features of a *novel TIMP-3* mutation causing Sorsby’s fundus dystrophy: Implications for disease mechanism. Br. J. Ophthalmol..

[26] Riera M., Navarro R., Ruiz-Nogales S., Mendez P., Bures-Jelstrup A., Corcostegui B., Pomares E. (2017). Whole exome sequencing using Ion Proton system enables reliable genetic diagnosis of inherited retinal dystrophies. Sci. Rep..

[27] Felbor U., Stohr H., Amann T., Schonherr U., Weber B.H. (1995). A novel Ser156Cys mutation in the tissue inhibitor of metalloproteinases-3 (TIMP3) in Sorsby’s fundus dystrophy with unusual clinical features. Hum. Mol. Genet..

[28] Lin R.J., Blumenkranz M.S., Binkley J., Wu K., Vollrath D. (2006). A novel His158Arg mutation in *TIMP3* causes a late-onset form of Sorsby fundus dystrophy. Am. J. Ophthalmol..

[29] Fung A.T., Stohr H., Weber B.H., Holz F.G., Yannuzzi L.A. (2013). Atypical sorsby fundus dystrophy with a novel tyr159cys timp-3 mutation. Retin. Cases Brief Rep..

[30] Felbor U., Suvanto E.A., Forsius H.R., Eriksson A.W., Weber B.H. (1997). Autosomal recessive Sorsby fundus dystrophy revisited: Molecular evidence for dominant inheritance. Am. J. Hum. Genet..

[31] Jacobson S.G., Cideciyan A.V., Bennett J., Kingsley R.M., Sheffield V.C., Stone E.M. (2002). Novel mutation in the *TIMP3* gene causes Sorsby fundus dystrophy. Arch. Ophthalmol..

[32] Weber B.H., Vogt G., Pruett R.C., Stohr H., Felbor U. (1994). Mutations in the tissue inhibitor of metalloproteinases-3 (*TIMP3*) in patients with Sorsby’s fundus dystrophy. Nat. Genet..

[33] Barbazetto I.A., Hayashi M., Klais C.M., Yannuzzi L.A., Allikmets R. (2005). A novel *TIMP3* mutation associated with Sorsby fundus dystrophy. Arch. Ophthalmol..

[34] Weber B.H., Vogt G., Wolz W., Ives E.J., Ewing C.C. (1994). Sorsby’s fundus dystrophy is genetically linked to chromosome 22q13-qter. Nat. Genet..

[35] Iyer P.G., Zhou H., Zhang Q., Chu Z., Shen M., Shi Y., Liu J., Trivizki O., Lam B.L., Wang R.K. (2022). Swept-Source Optical Coherence Tomography Detection of Bruch Membrane and Choriocapillaris Abnormalities in Sorsby Macular Dystrophy. Retina.

[36] Yannuzzi L.A., Rohrer K.T., Tindel L.J., Sobel R.S., Costanza M.A., Shields W., Zang E. (1986). Fluorescein angiography complication survey. Ophthalmology.

[37] Wang M., Gao S., Zhang Y., Zhang M. (2021). Sensitivity and specificity of optical coherence tomography angiography in the diagnosis of active choroidal neovascularization: A systematic review and meta-analysis. Graefes Arch. Clin. Exp. Ophthalmol..

[38] Arris C.E., Bevitt D.J., Mohamed J., Li Z., Langton K.P., Barker M.D., Clarke M.P., McKie N. (2003). Expression of mutant and wild-type TIMP3 in primary gingival fibroblasts from Sorsby’s fundus dystrophy patients. Biochim. Biophys. Acta.

[39] Langton K.P., Barker M.D., McKie N. (1998). Localization of the functional domains of human tissue inhibitor of metalloproteinases-3 and the effects of a Sorsby’s fundus dystrophy mutation. J. Biol. Chem..

[40] Langton K.P., McKie N., Smith B.M., Brown N.J., Barker M.D. (2005). Sorsby’s fundus dystrophy mutations impair turnover of TIMP-3 by retinal pigment epithelial cells. Hum. Mol. Genet..

[41] Soboleva G., Geis B., Schrewe H., Weber B.H. (2003). Sorsby fundus dystrophy mutation Timp3(S156C) affects the morphological and biochemical phenotype but not metalloproteinase homeostasis. J. Cell Physiol..

[42] Weber B.H., Lin B., White K., Kohler K., Soboleva G., Herterich S., Seeliger M.W., Jaissle G.B., Grimm C., Reme C. (2002). A mouse model for Sorsby fundus dystrophy. Investig. Ophthalmol. Vis. Sci..

[43] Rocholz R., Corvi F., Weichsel J., Schmidt S., Staurenghi G., Bille J.F. (2019). OCT Angiography (OCTA) in Retinal Diagnostics. High Resolution Imaging in Microscopy and Ophthalmology: New Frontiers in Biomedical Optics.

[44] Ensenberger M.G., Thompson J., Hill B., Homick K., Kearney V., Mayntz-Press K.A., Mazur P., McGuckian A., Myers J., Raley K. (2010). Developmental validation of the PowerPlex 16 HS System: An improved 16-locus fluorescent STR multiplex. Forensic Sci. Int. Genet..

[45] De Bonis P., Laborante A., Pizzicoli C., Stallone R., Barbano R., Longo C., Mazzilli E., Zelante L., Bisceglia L. (2011). Mutational screening of VSX1, SPARC, SOD1, LOX, and TIMP3 in keratoconus. Mol. Vis..

[46] Gelb B.D., Cave H., Dillon M.W., Gripp K.W., Lee J.A., Mason-Suares H., Rauen K.A., Williams B., Zenker M., Vincent L.M. (2018). ClinGen’s RASopathy Expert Panel consensus methods for variant interpretation. Genet. Med..

[47] Mester J.L., Ghosh R., Pesaran T., Huether R., Karam R., Hruska K.S., Costa H.A., Lachlan K., Ngeow J., Barnholtz-Sloan J. (2018). Gene-specific criteria for PTEN variant curation: Recommendations from the ClinGen PTEN Expert Panel. Hum. Mutat..

[48] Biesecker L.G., Harrison S.M., ClinGen Sequence Variant Interpretation Working G. (2018). The ACMG/AMP reputable source criteria for the interpretation of sequence variants. Genet. Med..

[49] Nagase H., Visse R., Murphy G. (2006). Structure and function of matrix metalloproteinases and TIMPs. Cardiovasc. Res..

[50] Li Z., Clarke M.P., Barker M.D., McKie N. (2005). *TIMP3* mutation in Sorsby’s fundus dystrophy: Molecular insights. Expert Rev. Mol. Med..

