# Supplementary material for: Disease-Causing TIMP3 Variants and Deep Phenotyping of Two Czech Families with Sorsby Fundus Dystrophy Associated with Novel p.(Tyr152Cys) Mutation
Source: Int J Mol Sci. 2024 Mar 27;25(7):3744. doi: 10.3390/ijms25073744 (PMC11011298; doi:10.3390/ijms25073744)
Supplement: Supplementary file 1 [file ijms-25-03744-s001.zip › ijms-2892729-supplementary.pdf]

SUPPLEMENTARY MATERIAL

Supplementary Table S1: Available evidence on variants reported as disease-causing in *TIMP3* (RefSeq NM_000362.5).

**Description**		**ACMG/AMP classification (criteria applied)**	**Description in the first report**		**Reported phenotype**	**Comment to changes in variant description compared to the original publication**	**Ref.**
**DNA**	**Protein**		**DNA level**	**Protein level**			
c.29T>A	p.(Leu10His)	Likely pathogenic (PM2_Moderate, PP3_Moderate, PS3_Supporting, PP4_Supporting, PP1_Supporting)	c.29T>A	p.(Leu10His)	Early-onset maculopathy without CNV		[16]
c.34G>C	p.(Gly12Arg)	Likely pathogenic (PM2_Moderate, PS3_Supporting, PP4_Supporting, PP1_Strong)	c.34G>C	p.(Gly12Arg)	Early-onset maculopathy without CNV		[16]
c.70T>C	p.(Cys24Arg)	Likely pathogenic (PM2_Moderate, PP3_Strong, PP4_Moderate)	Not provided	Cys24Arg	SFD (no clinical description)	No RefSeq or sequence trace image, however, all 5 variants in the original work correspond to NM_000362.5	[17]
c.113C>G	p.(Ser38Cys)	Pathogenic (PM2_Supporting, PS4_Strong, PP3_Moderate, PP4_Strong, PS3_Supporting, PP1_Strong)	Not provided	Ser38Cys	SFD		[17-20]
c.410A>G	p.(Tyr137Cys)	Likely Pathogenic (PM2_Moderate, PP3_Moderate, PM1_Moderate)	c.410A>G	p.Tyr137Cys	Retinitis pigmentosa-like, drusen	No RefSeq but UniProt ID provided	[21]
c.439-2dup	p.?	Likely Pathogenic (PM2_Moderate, PS4_Supporting, PP3_Supporting, PP4_Strong, PP1_Supporting)	A single base insertion at position –2 or –1 of exon 5 which converts CAG to CAAG at the 3' splice acceptor	Not provided	SFD	No RefSeq, variant description based on sequence gel image	[22]
c.452A>G	p.(Tyr151Cys)	Likely Pathogenic (PM2_Moderate, PP3_Supporting, PP4_Moderate, PM1_Moderate)	Not provided	Tyr151Cys	SFD (no clinical description)	No RefSeq or sequence trace image, however, all 5 variants in the original work correspond to NM_000362.5	[17]
c.455A>G	p.(Tyr152Cys)	Pathogenic (PM2_Moderate, PS4_Supporting, PP3_Supporting, PP4_Strong, PM1_Moderate, PM6_Moderate)	c.455A>G	p.(Tyr152Cys)	SFD		Current study
c.484G>A	p.(Glu162Lys)	Likely pathogenic (PM2_Moderate, PP3_Moderate, PP4_Strong, PS3_Supporting, PP1_Supporting)	c.415G>A	p.(E139K)	SFD	Variant description based on sequence trace image	[23]
c.484G>T	p.(Glu162*)	Likely pathogenic (PM2_Moderate, PP4_Strong, PS3_Supporting, PP1_Supporting)	Not provided	E139X	SFD	Variant description based on sequence trace image	[24,25]
c.499G>A	p.(Asp167Asn)	VUS (PM2_Moderate, PP3_Supporting, PP4_Moderate)	c.499G>A	Asp167Asn	MD or SFD (no clinical description)	No RefSeq or sequence trace image	[26]

c.521A>G	p.(Tyr174Cys)	Pathogenic (PM2_Moderate, PP3_Supporting, PP1_Supporting, PP4_Strong, PM1_Moderate)	c.452A>G	p.Tyr174Cys	SFD	No RefSeq or sequence trace image, variant description based on position of two other variants in the original work	[6]
c.530A>G	p.(Tyr177Cys)	Pathogenic (PM2_Moderate, PP3_Supporting, PP4_Strong, PM1_Moderate, PP1_Strong)	c.530A>G	p.Tyr177Cys	SFD	No RefSeq or sequence trace image	[6]
c.536C>G	p.(Ser179Cys)	Pathogenic (PM2_Moderate, PP3_Supporting, PP4_Strong, PS3_Supporting, PM1_Moderate, PP1_Strong)	Not provided	Ser156Cys	SFD	Variant description based on sequence trace image	[24,27]
c.542A>G	p.(His181Arg)	Pathogenic (PM2_Moderate, PP3_Supporting, PP4_Strong, PP1_Strong)	Not provided	His158Arg	SFD	Variant description based on sequence trace image	[28]
c.545A>G	p.(Tyr182Cys)	Pathogenic (PM2_Moderate, PP3_Supporting PP4_Strong, PM1_Moderate, PP1_Strong)	Not provided	Tyr159Cys	SFD	No RefSeq or sequence trace image, variant description based on a subsequent report	[6,29]
c.565G>T	p.(Gly189Cys)	Pathogenic (PM2_Moderate, PP3_Supporting PP4_Strong, PM1_Moderate, PS3_Supporting, PP1_Strong)	c.565G>T	p.(Gly166Cys)	SFD	Variant description based on sequence trace image	[24,30]
c.568G>T	p.(Gly190Cys)	Pathogenic (PM2_Moderate, PP3_Strong, PP4_Strong, PP1_Moderate)	The substitution of T for G in the first position of codon 167 of the *TIMP3* gene		SFD	No RefSeq or sequence trace image, variant description based on studies published at the time of the original report	[31]
c.572A>G	p.(Tyr191Cys)	Likely pathogenic (PM2_Moderate, PP3_Supporting, PP4_Strong, PM1_Moderate)	Not provided	Tyr168Cys	SFD	Variant description based on the sequence trace image	[32]
c.577A>T	p.(Ser193Cys)	Likely pathogenic (PM2_Moderate, PP3_Strong, PP4_Supporting, PP1_Strong)	c.508A>T	Ser170Cys	Retinitis pigmentosa-like, drusen	No RefSeq or sequence trace image, variant description based on studies published at the time of the original report	[33]
c.584A>G	p.(Tyr195Cys)	Pathogenic (PM2_Moderate, PP3_Strong, PP4_Strong, PP1_Supporting)	Not provided	Y172C	SFD	No RefSeq or sequence trace image, variant description based on studies published at the time of the original report	[31]
c.594G>T	p.(Trp198Cys)	Likely pathogenic (PM2_Moderate, PP4_Moderate, PM1_Moderate)	Not provided	Trp198Cys	SFD (no clinical description)	No RefSeq or sequence trace image, however, all 5 variants in the abstract correspond to NM_000362.5	[17]
c.610A>T	p.(Ser204Cys)	Pathogenic (PM2_Moderate, PP3_Supporting, PP4_Strong, PM1_Moderate, PP1_Strong)	Not provided	Ser181Cys	SFD	Variant description based on sequence trace image	[32,34]

Abbreviations: ACMG/AMP, The American College of Medical Genetics and Genomics/ Association for Molecular Pathology; RefSeq, NCBI Reference Sequence Database; VUS, variant of unknown significance; CNV, choroidal neovascular membrane; SFD, Sorsby fundus dystrophy; MD, macular dystrophy.

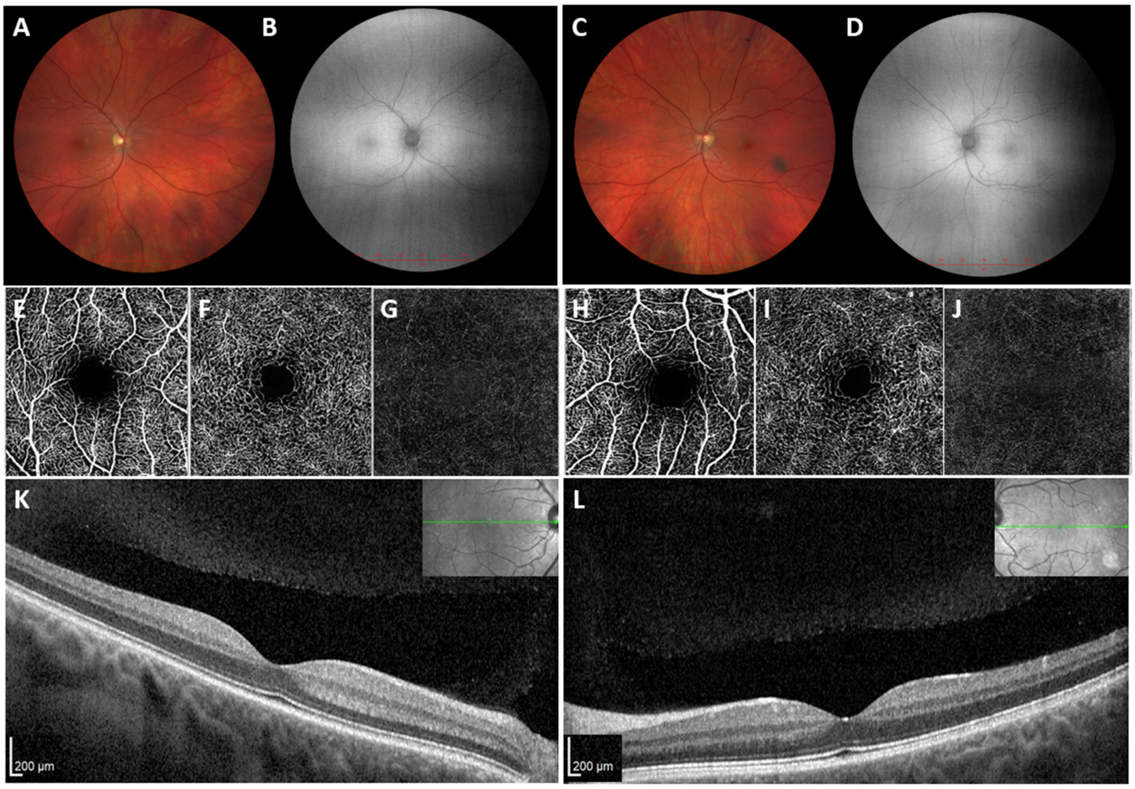

Supplementary Figure S1: Physiological SD-OCT and fundoscopic findings in a 30-year-old carrier of a pathogenic variant in *TIMP3* (right eye – left side, left eye – right side): (A, C) fundus photography, (B, D) FAF without a pathological signal pattern, (E through L) OCTA tomogram centred at the macula shows physiological vasculature bilaterally while (M, N) SD-OCT scans passing through foveolar area demonstrate normal architecture of the retinal layers in both eyes.

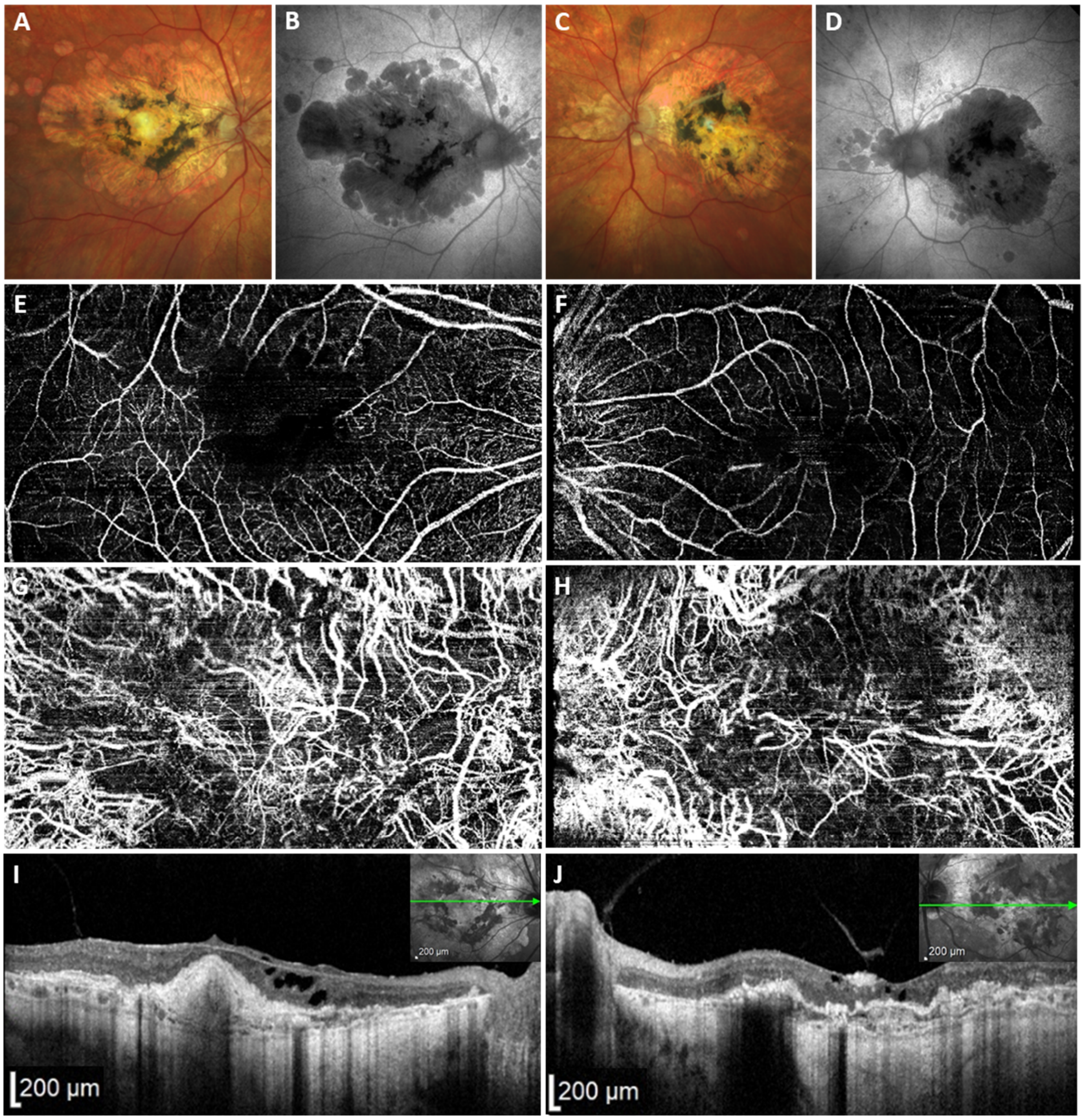

Supplementary Figure S2: Retinal imaging in individual II:1 from family 2. (A) Fundus photography of the right eye and (C) left eye demonstrates severe scarring and (B, D) atrophy predominantly in the macular region with a corresponding pattern of hypoautofluorescence. (E) OCTA scans of the macula demonstrate no lesion within the deep vascular complex in both the right and (F) left eye while the avascular complex layer is completely vascularized bilaterally (G, H). (I) Transversal SD-OCT scans show loss of the physiological architecture of the retinal layers in the right and (J) left eye, note severe scaring (indicated by arrows) and cystic changes (asterisks). Abbreviations: SD-OCT, spectral domain optical coherence tomography; OCTA, optical coherence tomography angiography.

Supplementary material:

List of genes screened in family 1

ABCA1 , ABCA4, ABCB6, ABHD12, ABHD5, ACBD5, ACO2, ACTB, ACTG1, ADAM9, ADAMTS10, ADAMTS17, ADAMTS18, ADGRV1, AGBL1, AGBL5, AGK, AGPS, AHI1, AIPL1, ALDH1A3, ALDH18A1, ALMS1, ANOS1, AP3B1, AP3D1, APC, ARHGEF18, ARL13B, ARL2BP, ARL6, ARMC9, ARNT2, ARR3, ARSG, ASB10, ATF6, ATOH7, ATP1A2, B3GALNT2, B3GALT1, B3GLCT, B3GNT1, BBS1, BBIP1, BBS2, BBS5, BCOR, BDNF, BEST1, BFSP1, BFSP2, BLOC1S3, BLOC1S6, BMP4, BRD4, B9D2,C12orf57, C1QTNF5, C21orf2, C2orf71, C8orf37, CA4, CABP4, CACNA1A, CACNA1F, CACNA2D4, CAPN5, CCDC111, CDH23, CDH3, CDHR1, CEP290, CEP78, CERKL, CFH, CFI, CIB2, CISD2, CLN3, CLRN1, CNGA1, CNGA3, CNGB1, CNGB3, CNNM4, COL11A1, COL11A2, COL18A1, COL25A1, COL2A1, COL4A1, COL8A2, COL9A1, COL9A2, COL9A3, COX7B, CPAMD8, CRB1, CRX, CRYAA, CRYAB, CRYBA1, CRYBA2, CRYBA4, CRYBB1, CRYBB2, CRYBB3, CRYGB, CRYGC, CRYGD, CRYGS, CTC1, CTNNA1, CTNNB1, CX3CR1, CYP1B1, CYP4V2, DAG1, DCN, DHDDS, DNM1L, DRAM2, DTNBP1, EDNRB, EFEMP1, ELOVL4, EPHA2, ELP4, EYA1, EYS, FAM126A, FAM161A, FBLN5, FBN1, FBN2, FKRP, FKTN, FLVCR1, FOXC1, FOXE3, FRMD7, FSCN2, FTL, FYCO1, FZD4, FZD5, GCNT2, GDF3, GDF6, GJA1, GJA3, GJA8, GLIS3, GMPPB, GNAT1, NAT2, GNB3, GPR143, GPR179, GRHL2, GRK1, GRM6, GSN, GUCA1A, GUCA1B, GUCY1A1, GUCY2D, GZF1, HARS, HCCS, HGSNAT, HK1, HMCN1, HMGB3, HMX1, HPS1, HPS3, HPS4, HPS5, HPS6, HS6ST2, HSF4, HTRA1, CHM, CHMP4B, CHN1, CHRDL1, CHST6, IDH3B, IDH3B, IFT140, IFT172, IFT27, IFT43, IMPDH1, IMPG1, IMPG2, INPP5E, IQCB1, ISPD, ITPR1, JAG1, KCNJ13, KCNV2, KERA, KIF11, KITLG, KIZ, KLHL7, KRT12, KRT3, LARGE, LCA5, LEMD2, LIM2, LMX1B, LOXL1, LRAT, LRIT3, LRMDA, LRP5, LRPAP1, LSS, LTBP2, LYST, MAB21L2, MAF, MAFB, MAK, MC1R, SNX3, MERTK, MFRP, MIP, MITF, MSTO1, MTPAP, MVK, MYO7A, MYOC, NAA10, NDP, NDUFB11, NDUFS1, NEK2, NHS, NHS, NMNAT1, NOTCH2, R2E3, R2F1, NRL, NTF4, NYX, OAT, OCA2, OFD1, OPA1, OPA3, OPN1LW, OPN1MW, OPN1SW, OPTN, OTX2, OVOL2, P3H2, P4HA2, PAX2, PAX3, PAX6, PCARE, PCDH15, PCYT1A, PDE6A, PDE6B, PDE6C, PDE6G, PDE6H, PDZD7, PEX26, PEX7, PHYH, PIK3R1, PIKFYVE, PITPNM3, PITX2,

PITX3, PLA2G5, PLK4, PMM2, POC1B, POLG, POMGNT1, POMGNT2, POMK, POMT1, POMT2, PORCN, PRCD, PRDM13, PRDM5, PROM1, PRPF3, PRPF31, PRPF4, PRPF6, PRPF8, PRPH2, PRPS1, PRSS56, PXDN, RAB18, RAB28, RAB3GAP1, RAB3GAP2, RARB, RAX, RAX2, RBP3, RBP4, RCBTB1, RD3, RDH11, RDH12, RDH5, REEP6, RGR, RGS9, RGS9BP, RHO, RIMS1, RLBP1, ROM1, RP1, RP1L1, RP2, RP9, RPE65, RPGR, RPGRIP1, RPGRIP1L, RS1, RTN4IP1, SAG, SALL2, SALL4, SCAPER, SCO2, SDCCAG8, SEMA4A, SH3PXD2B, SHH, SIPA1L3, SIX6, SLC16A12, SLC24A1, SLC24A5, SLC25A4, SLC38A8, SLC39A5, SLC45A2, SLC4A11, SLC7A14, SLITRK6, SMOC1, SNAI2, SNRNP200, SNX3, SOX1, SOX10, SOX2, SPATA7, SRD5A3, STRA6, TACSTD2, TBC1D20, TCF4, TDRD7, TEAD1, TEK, TENM3, TFAP2A, TGFBI, TIMP3, TINF2, TMEM126A, TMEM5, TMEM98, TOPORS, TRIM44, TRNT1, TRPM1, TSPAN12, TTC8, TTLL5, TUB, TUBGCP4, TUBGCP6, TULP1, TYR, TYRP1, UBIAD1, UNC119, UNC45B, USH1C, USH1G, USH2A, VAX1, VCAN, VIM, VSX1, VSX2, WDR36, WFS1, WHRN, WT1, XYLT2, YAP1, YME1L1, ZEB1, ZNF408, ZNF423, ZNF469, ZNF513, ZNF644, C5orf42, CASK, CC2D2A, CENPF, CEP104, CEP164, CEP41, CFAP410, CFB, CLDN19, COL17A1, CSPP1, CST3, CTDP1, CYP27A1, CYP51A1, DDX59, DHCR7, DNMBP, EED, EPG5, EPHA2, FAR1, FGF8, FGFR1, FLNA , FLN1), FOXD3, FRAS1, FREM1, FREM2, GALK1, GALT, GFER, GLI2, GLI3, GNPAT, GNPTG, GRIP1, GTF2H5, HESX1, HTRA2, HYLS1, CHD7, IARS2, INPP5K, ITM2B, JAM3, KIAA0586, KIF7, KIT, KMT2D, LAMB2, LCAT, LHX3, LHX4, LONP1, LOXL3, LRP2, LZTFL1, MAN2B1, MED25, MIPEP, MKKS, MKS1, MLPH, MSMO1, MYH9, MYO5A, NF2, NPHP1, NPHP3, NPHP4, OCRL, PANK2, PANK4, PEX1, PEX10, PEX11B, PEX12, PEX13, PEX14, PEX16, PEX19, PEX2, PEX3, PEX5, PEX6, PITX1, POLA1, POU1F1, PROKR2, ROP1, PTCH1, PUF60, RAB27A, SACS, SC5D, SCLT1, SETX, SIL1, SLC2A1, SLC33A1, SMO, SOX3, STN1, STS, STS, TAX1BP3, TGFBI, TLR4, TMEM237, TP63, TRAF3IP1, TRIM32, UBIAD1, VPS13B, WDPCP, WDR19, WRN, ZBTB20
